# Association Between Video-Derived Digital Operative Metrics and GEARS Scores in Robotic Surgery and Development of an Exploratory Multivariable Model

**DOI:** 10.3390/jcm15176786

**Published:** 2026-09-01

**Authors:** Xingyuan Xiao, Yuanzhuo Du, Zhenyu Liu, Xuewei Zhu, Kun Yang, Bing Li

**Affiliations:** 1 Department of Urology, Zhongnan Hospital of Wuhan University, Wuhan 430071, China; 2 Hubei Key Laboratory of Urological Diseases, Wuhan 430071, China; 3 Institute of Urology, Wuhan University, Wuhan 430071, China

**Keywords:** robotic surgery, GEARS, digital operative metrics, surgical skill assessment, video analysis, exploratory multivariable model

## Abstract

**Background**: Robotic-assisted surgery has been increasingly adopted across surgical specialties, but objective and efficient assessment of robotic surgical skills remains challenging. The Global Evaluative Assessment of Robotic Skills (GEARS) is commonly used for expert-based evaluation; however, it requires manual review and may not fully capture detailed operative processes. This study aimed to investigate the associations between video-derived digital operative metrics and expert-derived GEARS scores and to conduct an exploratory multivariable analysis of metrics associated with GEARS scores. **Methods**: Data were collected from 42 surgeons who completed a standardized robotic surgery training program. On the final training day, anonymized animal-operation videos were evaluated by blinded experts using the GEARS scale. Surgical videos were processed to extract 43 digital operative metrics related to instrument visibility, spatial positioning, camera control, clutch use, switching use, and motion characteristics. GEARS scores and digital operative metrics were compared according to annual laparoscopic surgical volume, including dichotomized volume groups, specialty-based subgroups, and volume tertiles. Stepwise multiple linear regression was used as an exploratory variable-selection approach to identify video-derived metrics associated with expert-derived GEARS scores. **Results**: After false discovery rate correction, no significant differences in GEARS scores or digital operative metrics were observed across annual laparoscopic surgical volume groups, specialty subgroups, or volume tertiles. The final regression model retained three predictors: mean camera-use count per minute, minimum distance from the left-side center point, and maximum distance from the right-side center point. The model had an apparent in-sample R^2^ of 0.509 (F = 9.588; *p* < 0.001). This value describes model fit within the present sample and has not been corrected for optimism. **Conclusions**: Video-derived digital operative metrics, particularly those reflecting camera control and instrument spatial distribution, were associated with expert-derived GEARS scores and may provide complementary quantitative information for expert-based robotic surgical skill assessment.

## 1. Introduction

Robotic-assisted surgery has been rapidly adopted across multiple surgical specialties, yet the objective and efficient assessment of robotic surgical skills remains challenging [1,2]. Although surgical volume and years of practice are commonly used as indicators of surgeon experience [3], these measures may not fully represent performance during a specific robotic procedure. Therefore, quantifiable process-based metrics are needed to complement conventional experience-based indicators and existing skill-assessment systems.

The Global Evaluative Assessment of Robotic Skills (GEARS) and the Objective Structured Assessment of Technical Skills (OSATS) are widely used tools for surgical skill assessment and have demonstrated standardization and clinical interpretability [4,5,6]. Nevertheless, these scoring systems largely depend on expert-based manual review, which is time-consuming and may be affected by observer bias and limited inter-rater consistency. A digital evaluation system using surgical video recordings can derive metrics including instrument coordinates, motion, pauses, clutch activations, and camera adjustments. These video-derived metrics may supplement conventional scoring systems by providing more granular, reproducible, and potentially automated process information, thereby facilitating more precise and efficient assessment [7,8]. However, it remains unclear whether video-derived digital operative metrics can provide an interpretable quantitative representation of expert-derived GEARS scores in a structured robotic surgery training setting.

Accordingly, this study aimed to evaluate the associations between video-derived digital operative metrics and expert-derived GEARS scores and to identify a combination of metrics associated with variation in GEARS scores using an exploratory multivariable analysis. We additionally examined whether annual laparoscopic surgical volume was associated with GEARS scores and digital operative metrics.

## 2. Materials and Methods

### 2.1. Subjects

Data were retrospectively collected from 42 surgeons who completed systematic training at the Surgical Robot Training Center of Wuhan University between February and April 2026. Participants were included in the present analysis if they completed the seven-day training program and the Day-7 assessment and had an analyzable operative video, a complete GEARS score, and complete digital operative metric data. Exclusion criteria were incomplete training, an incomplete or technically unusable video recording, or unavailable GEARS or digital operative metric data.

All 42 participants met these analytical criteria, and no participant was excluded from the final analysis. The cohort included 21 general surgeons, 15 urologists, and 6 gynecologists. Previous robotic surgical experience was not separately categorized as beginner, intermediate, or advanced in the original registration records. No financial or performance-based compensation was provided.

### 2.2. Training Protocol

**Figure 1:**
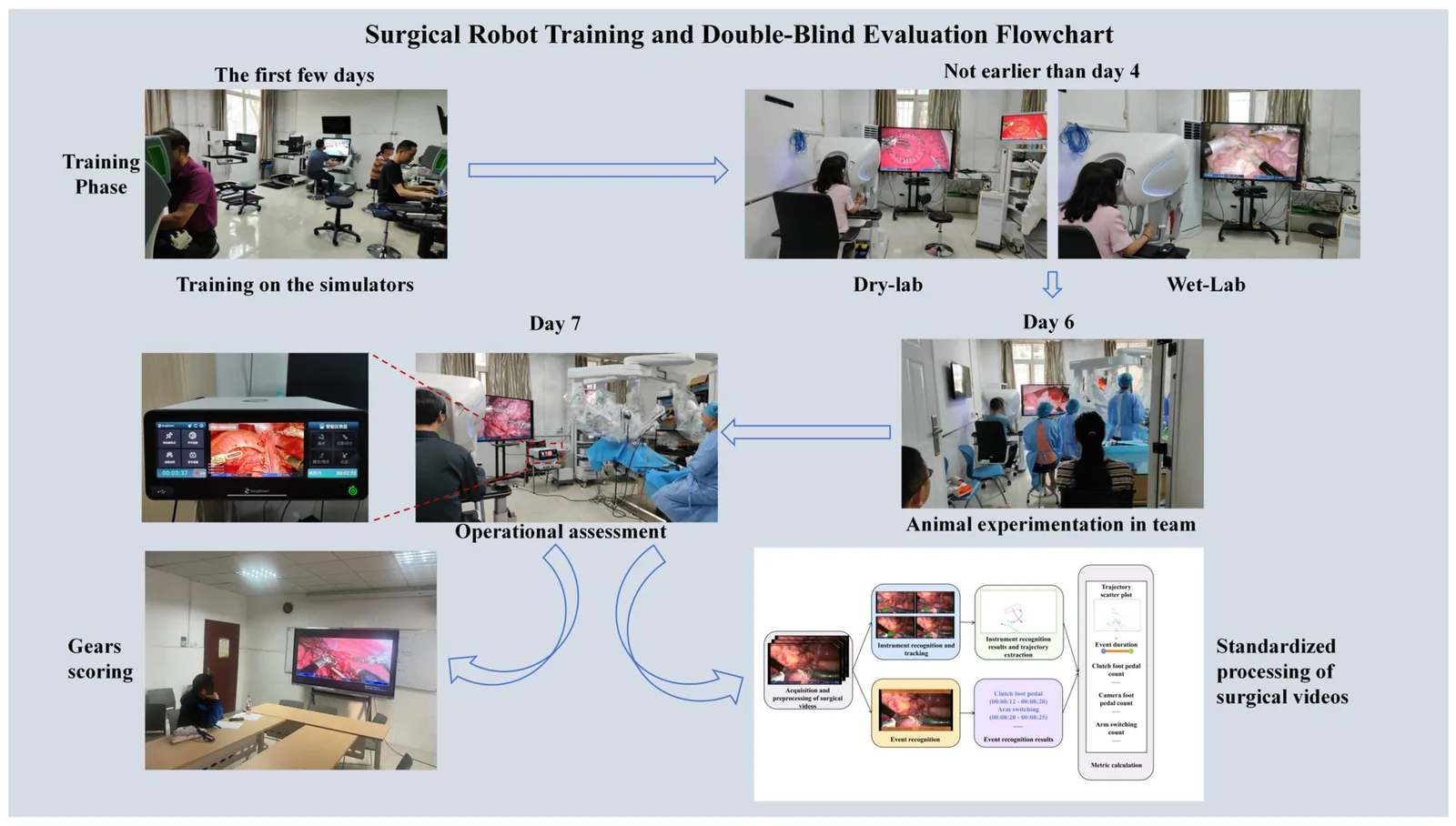
Flowchart of the training program.

The duration of each training cycle was 7 days. Basic and advanced simulator training was conducted using the dV-Trainer platform. Subsequent dry-laboratory, wet-laboratory, and animal-procedure training was conducted using the MP-1000 surgical robotic system (Jingfeng Medical, Shenzhen, China). During simulator training, participants progressed to the subsequent exercise after completing the assigned task according to the simulator’s built-in scoring system. A detailed description of the cross-specialty robotic surgery training and GEARS assessment protocol has been reported previously [9]. The curriculum is designed with a stepwise progression, sequentially comprising: basic simulator, advanced simulator, dry-lab on actual robot, wet-lab on actual robot, team wet-lab, team animal experiment, and team animal assessment. The first four days were individual sessions, which were completed independently by the trainees who served as primary surgeons. Starting from the morning of Day 5, the first assistants and primary surgeons in the team sessions were incorporated into the training, and the animal experiments on Days 6 and 7 were completed jointly. Animal experimental materials were Guangxi Bama miniature pigs weighing 42 kg, and the primary surgeons in each training session were a mix of doctors from various specialties. The animal experiment assessment procedures were divided by specialty: for general surgery, one or two of gastrectomy, cholecystectomy, intestinal anastomosis, and gastrointestinal anastomosis; for gynecology, pelvic lymphadenectomy and hysterectomy; and for urology, one or two of renal hilar dissection and ureteral transection-anastomosis (Figure 1).

### 2.3. Assessment Materials

(1) The registration forms of the trainees recorded data on gender, age, surgical experience, and specialty.

(2) On Day 7, the trainees performed the designated procedures for the animal experiment. Videos were recorded and personal information was removed.

(3) Each analyzed video represented one participant acting as the primary surgeon throughout the entire evaluated segment. Operative roles did not change during the segment, and no evaluated recording combined the performance of more than one participant. Each participant contributed one analyzed video and one participant-level observation. Although the procedures were conducted in a team setting, only the primary surgeon during the designated segment was assessed, and no evaluated operative team contributed recordings from multiple participants.

### 2.4. GEARS Scores

After every two training sessions, seven experts were randomly selected from a pool of twelve to independently assess the animal-procedure videos. All evaluators were experienced robotic surgeons who had each performed more than 150 robotic procedures during the preceding three years and more than 500 robotic procedures in total. All videos were anonymized before evaluation, and the evaluators were not provided with information regarding participant identity, specialty, or previous surgical experience.

(1) The experts went to assigned sites and performed the evaluations separately.

(2) During the assessment, the experts turned off their phones, the videos were played by designated staff, and each trainee was evaluated for at least 20 min. Evaluators were permitted to pause and replay video segments during scoring.

(3) The standard GEARS scale was used for assessment, with a total score of 30.

(4) Following the prespecified randomized, double-blind GEARS assessment protocol reported previously [9], the highest and lowest of the seven expert ratings were excluded for each participant, and the arithmetic mean of the remaining five ratings was used as the participant-level GEARS score. This aggregation rule was applied uniformly to all participants to limit the influence of an isolated unusually high or low rating.

Inter-rater reliability was assessed separately within each rating batch because evaluator composition differed across batches. Average-measures intraclass correlation coefficients with 95% confidence intervals were calculated for the seven expert ratings within each batch using a two-way random-effects model.

### 2.5. Technical Approach and Description for Operative Metric Acquisition

The analyzed surgical videos had a resolution of 1920 × 1080 pixels and a frame rate of 60 frames per second. All algorithms were developed and tested in Python 3.12 using Visual Studio Code. The deep-learning model was implemented using the PyTorch framework. OpenCV and NumPy were used for image and video processing, centroid extraction, and geometric transformation, whereas SciPy and Pandas were used for trajectory analysis and related calculations. Instrument detection and localization were performed using the RT-DETR architecture. The model-development dataset comprised 160,580 training samples, 20,060 validation samples, and 20,066 test samples. Model performance was evaluated using standard object-detection metrics. The model achieved an mAP@0.5 of 0.9421, an mAP@0.5:0.95 of 0.8185, a precision of 0.900, a recall of 0.901, and an F1 score of 0.901.

The robotic surgical videos were first standardized by converting the continuous surgical field videos into analyzable image frames in time sequence, and by unifying the frame rate, resolution, and image scale, to minimize variations in acquisition parameters across different sources. Subsequently, based on the trained instrument recognition model, the target instrument regions in each frame were automatically identified and localized to obtain their spatial positions in the surgical field. To further quantify the instrument motion characteristics, the two-dimensional coordinates of key instrument parts were extracted from the recognition results and continuously recorded in chronological video order, thereby generating instrument motion trajectories. From the trajectory data, we further calculated metrics including instrument displacement, motion velocity, range of motion, path stability, and operational rhythm. These metrics served to describe the spatiotemporal characteristics of instrument manipulation during robotic surgery and to offer quantitative support for subsequent evaluations of surgical skills, analysis of operation patterns, and human–robot collaboration studies.

To address temporary instrument occlusion, specular reflection, and detection loss, a multi-object tracking and dynamic-filtering framework was applied. RT-DETR bounding boxes were used between consecutive frames using IoU- and appearance-based information with Hungarian assignment. During short periods of detection loss, motion-state prediction was used to maintain the instrument trajectory and track identity, and feature matching was used to reconnect the trajectory after the instrument reappeared. A Savitzky–Golay filter was applied to the extracted centroid trajectories to reduce pixel-level localization noise and short-term tracking fluctuations while preserving rapid changes in velocity and acceleration. To reduce the influence of differences in image resolution, camera zoom, and field of view, instrument coordinates were normalized according to image dimensions and mapped into a dimensionless relative coordinate system. Some interface-related components of the current image-recognition pipeline were developed specifically for the Jingfeng robotic system.

Instrument velocity was calculated using a finite-difference method applied to the smoothed centroid trajectories. Motion stability was evaluated from trajectory variation and movement smoothness, including changes in acceleration over time. Operative rhythm was characterized using frequency-domain analysis of the continuous velocity sequence. Manual validation was performed using 100 one-minute video clips sampled from 20 surgical videos. Instrument trajectories predicted by the model were rendered and independently reviewed by two experienced surgical experts. Consensus judgments were used for the final accuracy assessment, which yielded a trajectory-validation accuracy of 94.81%.

### 2.6. Ethical Approval

This study was approved by the Hubei Provincial Center for Disease Control and Prevention, Hubei Academy of Preventive Medicine, Committee for the Management and Use of Laboratory Animals (approval number: 202660041). All human participants were informed of the study purpose, methods, potential risks and benefits prior to enrollment, and provided written informed consent voluntarily. During the experimental procedures, the Regulations on the Management of Laboratory Animals and the ARRIVE guidelines were strictly followed, and all measures were taken to minimize animal suffering and the number of animals used.

### 2.7. Data Analysis

All statistical analyses were conducted using SAS 9.4 software. Annual laparoscopic surgery volume was dichotomized at the median into two groups (<200 cases and ≥200 cases). Continuous variables with normal distribution were expressed as mean ± standard deviation, and intergroup comparisons were performed using an independent-samples *t*-test. Continuous variables with non-normal distribution were expressed as median (interquartile range) M (P25, P75), and comparisons were made using the Mann–Whitney U test, with Benjamini–Hochberg correction for false discovery rate (FDR) in multiple comparisons. Annual laparoscopic surgery volume was divided into three groups by tertiles (<153 cases, 154–246 cases, and ≥247 cases). The Jonckheere–Terpstra test was used to analyze the trend of indicators across groups, and Benjamini–Hochberg correction was applied for false discovery rate (FDR) in multiple comparisons. Using the GEARS scores of surgical videos as the dependent variable and all surgical operation indicators as independent variables, a stepwise multiple linear regression model was constructed. Stepwise regression was used as an exploratory variable-selection approach to identify a limited number of interpretable operative metrics. The coefficient of determination (R^2^) was used to evaluate the goodness of fit, and the F-test was employed to verify the overall significance of the model. The variance inflation factor (VIF < 5) was applied to indicate the absence of significant multicollinearity. Stepwise variable selection was based on the probability of F, with entry and removal criteria of *p* ≤ 0.05 and *p* ≥ 0.10, respectively. Stepwise regression was used as an exploratory variable-selection approach to identify a limited number of interpretable video-derived metrics. The retained variables were considered hypothesis-generating associations rather than validated predictors. The complete sequence of variable entry is presented in Supplementary Table S6. Linearity was assessed by examining scatterplots of each retained variable against the GEARS score. Residual normality and homoscedasticity were assessed by examining the residual distribution and residual-versus-fitted-value pattern. Influential observations were evaluated using standardized residuals, leverage values, and Cook’s distance. The independence of residuals was assessed using the Durbin–Watson statistic. As an exploratory sensitivity analysis addressing specialty-related heterogeneity, the stepwise regression analysis was repeated after excluding the specialty subgroup with the smallest sample size. The directions, magnitudes, 95% confidence intervals, and statistical significance of the regression coefficients were compared with those of the primary analysis. All 42 participants had complete GEARS scores and digital operative metric data; no missing data were present, and no imputation was performed. All tests were two-sided, and *p* < 0.05 was considered statistically significant.

## 3. Results

### 3.1. Baseline Characteristics of the Study Subjects

A total of 42 robotic surgical trainees were enrolled in this study and divided into a high-volume group (≥200 cases, *n* = 22) and low-volume group (<200 cases, *n* = 20) based on an annual laparoscopic surgery volume of 200 cases. The baseline characteristics of the two groups are presented in Table 1. Demographic characteristics: The age distribution in both groups was non-normal. The overall age was 41.50 (38.00, 48.25) years, with 41.00 (38.75, 47.25) years in the high-volume group and 42.00 (37.00, 51.75) years in the low-volume group. Overall, 85.7% (36/42) of the participants were male, with 72.7% (16/22) in the high-volume group and 100% (20/20) in the low-volume group. Surgically related characteristics: The years of laparoscopic surgery experience were normally distributed, with an overall mean of 11.76 ± 4.63 years, 13.00 ± 4.15 years in the high-volume group, and 10.40 ± 4.85 years in the low-volume group. The total number of laparoscopic surgeries was non-normally distributed, with an overall median of 2220 (1180.00, 3540.00) cases, 3180.00 (2360.00, 4300.00) in the high-volume group, and 1160.00 (772.50, 1665.00) in the low-volume group. Regarding specialty and procedure distribution: general surgery was the most common specialty (50.0%, 21/42), followed by urology (35.7%, 15/42) and gynecology (14.3%, 6/42); all gynecologists were concentrated in the high-volume group (6/22, 27.3%), while none were in the low-volume group. The most frequently performed procedure was cholecystectomy plus intestinal anastomosis (31.0%, 13/42), followed by renal hilar dissection plus ureteral anastomosis (28.6%, 12/42).

Batch-specific inter-rater reliability was assessed for the expert-derived GEARS ratings. The average-measures ICC was 0.601 (95% CI, 0.250–0.841) for Batch 1, 0.709 (95% CI, 0.421–0.887) for Batch 2, and 0.638 (95% CI, 0.251–0.865) for Batch 3. The complete results are presented in Supplementary Table S5.

### 3.2. Comparison of Indicators Between Groups Stratified by Annual Laparoscopic Surgery Volume (200 Cases)

The 43 surgical operation metrics and GEARS scores were compared between the two groups stratified by annual laparoscopic surgery volume (<200 vs. ≥200 cases). Using an independent-samples *t*-test for normally distributed variables or Mann–Whitney U test for non-normally distributed variables, with Benjamini–Hochberg correction for FDR, no significant differences were found in any of the metrics (all *p* > 0.05 after FDR correction) (Table 2).

### 3.3. Subgroup Analyses by Specialty

Based on the overall null findings, subgroup analyses were further conducted by specialty (urology, general surgery, and gynecology). The results showed that within each specialty subgroup, no statistically significant differences were observed in surgical operation metrics or GEARS scores between the <200 and ≥200 groups (FDR > 0.05), consistent with the overall analysis, indicating good robustness of the results (Table 3). Since all gynecology trainees had annual volumes ≥200 cases, subgroup analysis was not performed for this specialty.

### 3.4. Trend Analysis by Tertiles of Annual Laparoscopic Surgery Volume

To further explore the association between surgical volume and the metrics, annual laparoscopic surgery volume was divided into tertiles (<153 cases, 154–246 cases, and ≥247 cases). The Jonckheere–Terpstra test was used to analyze the trend of metrics across the increasing volume groups. The results showed that neither GEARS scores nor any of the surgical operation metrics exhibited a significant linear trend across the volume groups (low to high) (FDR > 0.05). Although a modest decreasing trend was observed in certain metrics (duration with one robotic surgical instrument visible on the right side: Z = −2.330, *p* = 0.020), following FDR adjustment, the adjusted *p*-value was 0.655, indicating no statistically significant difference (Table 4).

### 3.5. Exploratory Multivariable Analysis of GEARS Scores

Because the sample sizes for individual procedures were insufficient for reliable procedure-specific modeling, a pooled exploratory analysis was performed. All participants completed the same structured training program, were assessed at the same final training stage using the same GEARS framework, and underwent the same video-processing pipeline. However, pooling did not imply that the different operative procedures were technically equivalent, and residual effects of specialty and procedure type could not be excluded. A stepwise multiple linear regression was then performed with the GEARS score as the dependent variable and the 43 operative metrics as candidate-independent variables. The results showed that the fourth step of the stepwise procedure included three retained metrics: mean camera-use count per minute, minimum distance from the left-side center point, and maximum distance from the right-side center point. The exploratory model had an apparent in-sample R^2^ of 0.509, and the overall F test was statistically significant (F = 9.588; *p* < 0.001). The R^2^ has not been corrected for optimism and describes model fit within the present sample only. Collinearity diagnostics showed that the variance inflation factor (VIF) for all three indicators was below five, indicating no substantial multicollinearity among these variables. The three retained metrics showed the following associations with expert-derived GEARS scores: mean camera-use count per minute was positively associated with GEARS scores (B = 0.863), minimum distance from the left-side center point was positively associated with GEARS scores (B = 104.389), and maximum distance from the right-side center point was negatively associated with GEARS scores (B = −17.074). Detailed regression coefficients and the sample-specific fitted regression equation are provided in Supplementary Table S4. The Durbin–Watson statistic was 2.183, indicating no evidence of substantial first-order autocorrelation in the residuals. The complete variable-entry sequence and the corresponding unstandardized coefficients, standard errors, standardized coefficients, 95% confidence intervals, *p*-values, and VIF values are presented in Supplementary Table S6. Diagnostic assessment suggested approximately linear relationships between the retained variables and GEARS scores, an approximately normal residual distribution, and no clear evidence of substantial heteroscedasticity (Supplementary Figure S1). No observation showed evidence of undue influence based on standardized residuals, leverage values, or Cook’s distance.

After exclusion of the smallest specialty subgroup, the directions and approximate magnitudes of the coefficients for the three principal video-derived metrics were broadly similar to those in the primary analysis. Mean camera-use count per minute remained statistically significant (B = 0.840, 95% CI 0.307–1.373; *p* = 0.003), whereas minimum distance from the left-side center point (*p* = 0.064) and maximum distance from the right-side center point (*p* = 0.083) did not reach statistical significance. These findings indicated broadly consistent association directions but reduced statistical precision in the smaller sensitivity-analysis sample (Supplementary Table S7).

## 4. Discussion

This study conducted an exploratory multivariable analysis of the associations between video-derived operative metrics and expert-derived GEARS scores. Unlike traditional global scoring based solely on expert review, the model examined whether quantitative parameters related to camera control, instrument spatial positioning, and motion trajectories could account for variation in expert-derived GEARS scores. The model therefore represents an exploratory link between video-derived process metrics and expert ratings rather than a direct measure of clinical operative quality. The exploratory model had an apparent in-sample R^2^ of 0.509. Because the analysis was not internally validated, this estimate may be optimistic and should not be interpreted as out-of-sample predictive performance. The specialty-exclusion sensitivity analysis showed broadly similar coefficient directions; however, only mean camera-use count per minute remained statistically significant, indicating that the estimates for the spatial metrics were less precise after reducing the sample size. These findings indicate that certain digital metrics, especially those related to camera manipulation and instrument spatial distribution, were associated with expert assessments and may reflect aspects of surgical field visualization, spatial positioning, and bilateral instrument coordination. The specific contribution of the present study is the identification of a small number of directly interpretable video-derived metrics that link camera control and instrument spatial positioning with variation in expert-derived GEARS scores.

These findings support the potential role of digital metrics in the development of automated scoring models for robotic surgical skills. The GEARS and OSATS are classic tools in surgical skill evaluation, and previous studies have demonstrated their reliability and discriminative ability in surgical skill assessment from the perspectives of scale development, external validation, and construct validity. Goh et al. developed and validated the GEARS scale and demonstrated its ability to distinguish different levels of robotic surgical skill [4]. Aghazadeh et al. and Sánchez et al. further confirmed the discriminative validity of GEARS in robotic surgery training settings [5,10]. Martin et al. and Faulkner et al. also demonstrated that OSATS can reliably and validly assess surgical skills [6,11]. The above findings indicate that the selection of GEARS as the dependent variable in our exploratory multivariable model was not arbitrary, but rather grounded in good clinical interpretability and a solid evaluation framework. Therefore, this study does not directly negate expert-based rating scales; instead, on the basis of existing well-established scoring systems, it further examines whether digital metrics can explain part of the information reflected in expert assessments.

The present study showed an apparent in-sample model fit corresponding to an R^2^ of 0.509. This suggests that their value may extend beyond descriptive characterization of the operative process and that they may have quantifiable associations with established expert-based assessments. From an operative perspective, the three variables retained in the model have plausible process-based interpretations. The positive coefficient for mean camera-use count per minute may reflect active adjustment of field exposure, viewing angle, and visual anticipation during task progression. Within the observed range, purposeful camera adjustment may contribute to maintenance of an appropriate operative view. However, more frequent camera use should not necessarily be interpreted as better performance, because excessive adjustment may also indicate inadequate initial positioning, uncertainty regarding exposure, or interruption of operative flow. The positive coefficient for minimum distance from the left-side center point may reflect broader but controlled use of the left-sided workspace, whereas the negative coefficient for maximum distance from the right-side center point may suggest that excessive right-sided excursion is associated with less efficient workspace control. These interpretations are not causal, and the spatial metrics may also have been influenced by anatomy, port placement, procedure type, operative-field size, and instrument configuration. Previous studies support this interpretation. Oh et al. [7] compared GEARS with objective performance indicators (OPIs) in robotic thoracic surgery and reported that OPIs can be generated from system events, console activity, and instrument kinematic data, thereby providing objective process information for skill assessment. These findings demonstrate that objective operative data can be compared with and complement expert ratings such as GEARS, which supports the rationale of the present study to use digital metrics to interpret GEARS scores. Chen et al. [12] used automated performance metrics (APMs) to evaluate robotic bladder-urethral anastomosis performance and provide training feedback, indicating that automated metrics can not only record operative performance but also further serve training feedback. Hung et al. further combined APMs with machine learning to assess performance in robot-assisted prostatectomy and predict outcomes [13,14]. Their validation studies on objective performance indicators also showed that robotic operation metrics in specific surgical steps could correspond to expert evaluations [15]. Therefore, the objective metrics extracted from the robotic system and surgical videos are not merely isolated technical parameters, but rather process-based information that may reflect aspects of performance captured by expert-derived GEARS ratings.

Meanwhile, Dubin et al. examined subjective and objective scoring in robotic simulators and suggested that different scoring systems may capture distinct dimensions of skill information [16]. Guni et al. also found that a task-specific checklist could complement GEARS in robotic suturing assessment [17]. These studies further indicate that digital metrics and GEARS should not be viewed as overlapping constructs. GEARS reflects the expert’s global judgment of robotic surgical performance, whereas digital metrics capture specific process-level features such as camera control, instrument motion, clutch usage, and motion trajectories. When combined, the two approaches can complement each other by bridging global assessment and process explanation. Therefore, this study is not a simple comparison between human ratings and machine-derived metrics, but rather an attempt to establish an interpretable bridge between them.

The present study also found no consistent association between annual laparoscopic surgery volume and either GEARS scores or the 43 digital operative metrics. Whether annual volume was dichotomized at 200 cases, categorized by tertiles, or analyzed in specialty-based subgroups, none of the associations remained statistically significant after false discovery rate correction. These findings do not imply that annual laparoscopic surgery volume is without value. Rather, they suggest that, in the present study population, annual laparoscopic surgery volume may not be a sufficiently sensitive indicator for distinguishing expert-rated robotic surgical performance. One possible explanation is that most surgeons included in this study already had substantial laparoscopic experience and relatively mature fundamental surgical skills. The overall difference associated with increasing annual laparoscopic surgery volume may not be pronounced, a finding also suggested by previous studies. Bravi et al., in a study of the learning curve for robot-assisted radical prostatectomy, suggested that the maturation of robotic surgical skill may be influenced by training systems, case mix, and individual variability [18]. Addison et al., in a video-based assessment of robotic bariatric surgery, found that surgeon experience was associated only with selected GEARS domains and operative time, rather than showing a consistent association with the total GEARS score [19]. Aghazadeh et al. further demonstrated that performance on robotic simulation tasks was associated with clinical robotic surgical performance, although this relationship depended on the specific task and assessment tool used [20]. Therefore, this suggests that surgeon experience is not a single variable that can be fully equated with surgical quality. Annual laparoscopic surgery volume may serve as a reference for surgeon background and experience accumulation, but it cannot fully account for specific operative performance in robotic surgery. Annual case volume also does not fully capture the recency, complexity, or quality of practice, and robotic surgical learning may be nonlinear and task-specific. Therefore, surgical volume should be regarded as a background indicator of accumulated experience rather than a direct substitute for contemporaneous performance assessment or as the sole basis for credentialing.

Another notable finding was that the group-based analyses did not identify statistically significant differences, whereas the fitted model identified a multivariable association with GEARS scores. These findings are not necessarily contradictory because the two approaches address different questions. Group comparisons evaluate average differences between predefined annual-volume categories and may lose information through categorization, whereas the regression model evaluates combinations of continuous operative features at the individual level. Previous studies have also shown that video-derived and kinematic features can be used to develop surgical skill assessment models. Lee et al. [21] used deep learning for multi-instrument tracking in robotic surgical skill evaluation, demonstrating that instrument trajectories and video information can reflect operator performance. Wang et al. [22] employed a multi-task convolutional neural network to assess robotic surgical training videos and predict GEARS scores, further supporting that a model-based relationship can be established between video-derived features and expert ratings. Jarc et al. [23] demonstrated that camera control metrics can serve as objective performance indicators in robotic surgery, which is highly consistent with the inclusion of “average number of camera manipulations per minute” in our model. Li et al. [24] proposed a cross-platform method for robotic surgical skill assessment based on clutch usage, suggesting that specific operative events on the robotic platform can also serve as a basis for skill evaluation. Compared with previous approaches based on high-dimensional machine-learning features or system-generated kinematic data, the present analysis focused on a limited number of directly interpretable video-derived metrics related to camera control and instrument spatial positioning. If validated in larger and more homogeneous datasets, video-derived metrics may have potential applications in robotic surgical training. Metrics related to camera use, workspace utilization, and instrument trajectories could be incorporated into individualized feedback, performance benchmarks, dashboard-based visualization, or longitudinal training records. Such applications may help identify specific process-level behaviors that are not apparent from a single global score.

However, this study has several limitations. First, the sample size was limited, particularly after stratification into three volume groups and specialty-based subgroup analyses, which reduced statistical power. Penalized regression methods, such as LASSO or elastic-net regression, may provide more stable variable selection and should be considered in future studies with larger datasets and appropriate internal and external validation. Second, there was heterogeneity in surgical procedures and specialties among the study participants. Procedural heterogeneity may have influenced the digital metrics independently of surgical skill and may have introduced residual confounding into the pooled model. Digital metrics may have been influenced by procedural complexity, video quality, the number of instruments used, and differences in operative tasks. Third, the current prediction formula remains exploratory and has not undergone formal internal or external validation; therefore, it cannot directly replace GEARS scoring or be used for individual assessment. Fourth, the linear regression model may not capture nonlinear relationships or interactions among operative behaviors. Fifth, the sample-specific fitted equation has not undergone formal internal or external validation and has not been corrected for optimism. Therefore, the apparent in-sample R^2^ may be optimistic, and the equation should not be used for individual-level prediction or application outside the present dataset. Because this was a single-center study using animal procedures, the findings cannot be assumed to generalize directly to human clinical surgery, other institutions, other procedures, or other robotic platforms. Instrument recognition and trajectory extraction may also have been affected by occlusion, glare, image noise, lighting variation, and temporary tracking loss. In addition, because evaluator composition differed across rating batches, inter-rater reliability was estimated separately within each batch rather than across the full cohort. Although the batch-specific average-measures ICCs provided evidence of rating reliability, their confidence intervals were relatively wide because each batch included only 14 participants. Measurement variability in the expert-derived GEARS ratings therefore cannot be completely excluded. Future studies should expand the sample size, incorporate standardized operative tasks and multicenter datasets, and conduct formal internal and external validation to assess the stability and generalizability of the model.

In conclusion, this study identified exploratory associations between video-derived digital operative metrics and expert-derived GEARS scores. Metrics related to camera control and instrument spatial positioning showed consistent associations with GEARS scores in the exploratory analysis. Annual surgical volume did not consistently distinguish GEARS scores or digital operative metrics in this study population. These findings support the potential value of video-derived metrics as quantitative adjuncts to expert-based assessment, while further validation in larger and more standardized datasets is required.

## 5. Conclusions

This study identified associations between video-derived digital operative metrics and expert-derived GEARS scores. Metrics related to camera control and instrument spatial positioning were retained in an exploratory multivariable analysis, whereas annual laparoscopic surgical volume did not consistently distinguish GEARS scores or digital operative metrics. These findings support the potential value of video-derived metrics as quantitative adjuncts to expert-based assessment. However, the sample-specific fitted equation was not internally validated and should not be used for individual-level prediction without further validation.

## Figures and Tables

**Table 1:** Baseline characteristics.

Variables	Total (*n* = 42)	High-Volume Group (≥200, *n* = 22)	Low-Volume Group (<200, *n* = 20)
Age	41.50 (38.00, 48.25)	41.00 (38.75, 47.25)	42.00 (37.00, 51.75)
Gender			
Male	36 (85.7)	16 (72.7)	20 (100)
Female	6 (14.3)	6 (27.3)	0 (0)
Laparoscopic surgery experience (years)	11.76 ± 4.63	13.00 ± 4.15	10.40 ± 4.85
Total laparoscopic volume (*n*)	2220 (1180.00, 3540.00)	3180.00 (2360.00, 4300.00)	1160.00 (772.50, 1665.00)
Specialty			
Gynecology	6 (14.3)	6 (27.3)	0 (0)
Urology	15 (35.7)	5 (22.7)	10 (50.0)
General surgery	21 (50.0)	11 (50.0)	10 (50.0)
Surgical procedure			
Intestinal anastomosis	2 (4.8)	1 (4.5)	1 (5.0)
Cholecystectomy + intestinal anastomosis	13 (31.0)	7 (31.8)	6 (30.0)
Renal hilar dissection	2 (4.8)	1 (4.5)	1 (5.0)
Renal hilar dissection + ureteral transection-anastomosis	12 (28.6)	3 (13.6)	9 (45.0)
Gastrointestinal anastomosis	1 (2.4)	0 (0)	1 (5.0)
Ureteral transection-anastomosis	1 (2.4)	1 (4.5)	0 (0)
Gastrointestinal anastomosis + intestinal anastomosis	1 (2.4)	1 (4.5)	0 (0)
Gastrectomy	2 (4.8)	2 (9.1)	0 (0)
Gastrectomy + intestinal anastomosis	2 (4.8)	0 (0)	2 (10.0)
Hysterectomy + pelvic lymphadenectomy	6 (14.3)	6 (27.3)	0 (0)
Operative time (seconds)	5997.57 ± 3608.74	5376.82 ± 3199.94	6680.40 ± 3980.93

Non-normally distributed data are presented as median (interquartile range). [x ± s; *n*, %; M (P25, P75)].

**Table 2:** Comparison of relevant metrics in a robotic training system across groups stratified by annual laparoscopic surgery volume.

Robotic Training System Metrics	Annual Laparoscopic Surgery Volume ≥ 200 (*n* = 22)	Annual Laparoscopic Surgery Volume < 200 (*n* = 20)	*p*-Value	FDR
GEARS score based on surgical video recordings	24.4140 ± 1.8576	24.6950 ± 1.2292	0.563	0.808
Minimum distance from the left-side center point	0.0034 (0.0019, 0.0081)	0.0033 (0.0018, 0.0060)	0.696	0.881
Minimum distance from the right-side center point	0.0041 (0.0021, 0.0090)	0.0033 (0.0020, 0.0057)	0.385	0.808
Total number of instrument-out-of-view events	330.5900 ± 224.7600	449.0500 ± 319.3180	0.169	0.808
Duration with one robotic surgical instrument visible on the left side	3849.9995 ± 2139.2070	5101.8880 ± 2880.8973	0.116	0.808
Duration with two robotic surgical instruments visible on the left side	551.4550 (221.8700, 1088.5125)	483.7200 (188.2475, 698.5625)	0.597	0.808
Duration with three robotic surgical instruments visible on the left side	16.9500 (0.0000, 56.4925)	5.8150 (2.7800, 11.2425)	0.464	0.808
Mean duration per minute with three robotic surgical instruments visible on the left side	0.1509 (0.0000, 0.5278)	0.0565 (0.0276, 0.1402)	0.479	0.808
Duration with one robotic surgical instrument visible on the right side	2324.6650 (1303.3225, 3038.1175)	3862.7850 (1713.6625, 5319.9775)	0.041	0.808
Mean duration per minute with two robotic surgical instruments visible on the right side	19.6594 ± 12.7436	12.7842 ± 8.3470	0.044	0.808
Total clutch-use count	39.5000 (7.0000, 72.2500)	51.0000 (10.2500, 109.0000)	0.392	0.808
Total camera-use duration	155.3850 (43.7675, 243.6300)	253.0350 (67.7200, 436.2650)	0.190	0.808
Mean camera-use count per minute	0.6550 (0.4025, 0.8775)	0.6650 (0.5125, 1.2100)	0.554	0.808
Total switching-use count	34.0000 (3.7500, 50.2500)	33.0000 (2.7500, 53.7500)	0.990	0.990
Total switching-use duration	167.5550 (23.8375, 275.1775)	169.5200 (22.6100, 259.4100)	0.743	0.884

Detailed summary table available in Supplementary Table S1. FDR: false discovery rate. All time-related metrics in the table are reported in seconds. The operative field was divided equally into left and right sides, and spatial distances were calculated with respect to the centers of the left and right halves.

**Table 3:** Comparison of operative performance metrics in a robotic training system between urology and general surgery groups stratified by annual laparoscopic surgery volume.

Robotic Training System Metrics	Urology		*p*-Value	FDR	General Surgery		*p*-Value	FDR
	Annual Laparoscopic Surgery Volume ≥ 200	Annual Laparoscopic Surgery Volume < 200			Annual Laparoscopic Surgery Volume ≥ 200	Annual Laparoscopic Surgery Volume < 200		
GEARS score based on surgical video recordings	26.5250 ± 1.0935	24.7600 ± 1.1108	0.019	0.116	23.7670 ± 1.9251	24.6300 ± 1.3953	0.251	0.757
Minimum distance from the left-side center point	0.0098 ± 0.0079	0.0048 ± 0.0030	0.294	0.404	0.0029 (0.0015, 0.0048)	0.0027 (0.0016, 0.0051)	0.717	0.837
Minimum distance from the right-side center point	0.0116 (0.0073, 0.0153)	0.0047 (0.0026, 0.0072)	0.048	0.162	0.0025 (0.0010, 0.0042)	0.0020 (0.0009, 0.0041)	0.621	0.837
Total number of instrument-out-of-view events	110.7500 ± 68.3930	335.4000 ± 190.0900	0.043	0.158	436.3300 ± 227.6780	562.7000 ± 387.8400	0.353	0.822
Duration with one robotic surgical instrument visible on the left side	1192.9275 ± 501.8955	4740.7940 ± 2437.4942	0.015	0.110	4676.6617 ± 1799.0072	5462.9820 ± 3360.0867	0.518	0.837
Duration with two robotic surgical instruments visible on the left side	107.2275 ± 64.1984	438.5650 ± 248.3653	0.002	0.088	691.2050 (399.9200, 2061.1025)	463.8900 (181.9675, 1067.5825)	0.187	0.757
Duration with three robotic surgical instruments visible on the left side	0.0000 (0.0000, 0.9975)	4.5100 (2.6050, 8.8875)	0.004	0.088	22.5650 (16.2400, 182.6800)	6.6650 (2.5275, 73.5300)	0.209	0.757
Mean duration per minute with three robotic surgical instruments visible on the left side	0.0000 (0.0000, 0.0278)	0.0617 (0.0412, 0.1291)	0.011	0.106	0.2576 (0.1507, 1.3934)	0.0500 (0.0200, 0.8838)	0.234	0.757
Duration with one robotic surgical instrument visible on the right side	939.9775 ± 360.2780	3671.9500 ± 1784.1713	0.012	0.106	3242.2467 ± 1550.0826	4034.7530 ± 2802.0157	0.411	0.822
Mean duration per minute with two robotic surgical instruments visible on the right side	3.2206 (2.0728, 13.1370)	10.9626 (2.4754, 13.9908)	0.572	0.680	21.5060 ± 11.8231	16.0553 ± 8.4472	0.237	0.757
Total clutch-use count	6.2500 ± 0.9570	40.7000 ± 38.9760	0.021	0.116	63.4200 ± 49.2720	134.5000 ± 138.1720	0.150	0.757
Total camera-use duration	24.5375 ± 9.3376	257.9700 ± 232.8841	0.011	0.106	217.6150 (126.7350, 259.0900)	306.0550 (45.2950, 586.1850)	0.598	0.837
Mean camera-use count per minute	0.5625 ± 0.3531	1.076 ± 0.8557	0.277	0.397	0.5500 (0.3800, 0.9575)	0.6200 (0.4775, 1.0675)	0.692	0.837
Total switching-use count	0.0000 (0.0000, 4.5000)	30.5000 (6.0000, 35.2500)	0.030	0.132	40.6700 ± 20.7290	40.4000 ± 36.4300	0.984	0.984
Total switching-use duration	0.0000 (0.0000, 32.3250)	135.1400 (25.8675, 210.6650)	0.043	0.158	240.2600 ± 133.0020	193.3140 ± 160.3762	0.461	0.837

Detailed summary table available in Supplementary Table S2. FDR: false discovery rate. All time-related metrics in the table are reported in seconds. The operative field was divided equally into left and right sides, and spatial distances were calculated with respect to the centers of the left and right halves.

**Table 4:** Trend analysis of operative performance metrics in a robotic training system by annual laparoscopic surgery volume.

Robotic Training System Metrics	Annual Laparoscopic Surgery Volume <153	Annual Laparoscopic Surgery Volume (154–246)	Annual Laparoscopic Surgery Volume ≥247	Z Score	*p*-Value	FDR
GEARS score based on surgical video recordings	24.8000 (23.8750, 25.2000)	25.0000 (22.3250, 25.6500)	25.0000 (23.3000, 25.8000)	0.393	0.695	0.910
Minimum distance from the left-side center point	0.0040 (0.0018, 0.0053)	0.0027 (0.0014, 0.0055)	0.0048 (0.0020, 0.0116)	0.993	0.321	0.733
Minimum distance from the right-side center point	0.0026 (0.0018, 0.0052)	0.0040 (0.0019, 0.0078)	0.0046 (0.0023, 0.0090)	1.224	0.221	0.710
Total number of instrument-out-of-view events	408.0000 (236.7500, 691.0000)	320.5000 (180.5000, 575.2500)	296.5000 (104.5000, 417.7500)	−1.523	0.128	0.655
Duration with one robotic surgical instrument visible on the left side	5720.7500 (4006.8450, 7392.3175)	4582.4650 (1365.4950, 7112.0825)	3656.3200 (1455.3200, 5147.5475)	−1.915	0.055	0.655
Duration with two robotic surgical instruments visible on the left side	579.0500 (201.4175, 715.5550)	422.5850 (146.7225, 1438.3400)	428.6000 (221.8700, 775.9425)	−0.046	0.963	0.963
Duration with three robotic surgical instruments visible on the left side	6.6650 (2.8650, 10.3675)	11.6850 (2.2300, 78.4450)	13.7300 (0.0000, 44.5675)	0.602	0.547	0.860
Mean duration per minute with three robotic surgical instruments visible on the left side	0.0503 (0.0222, 0.0741)	0.1482 (0.0294, 0.7671)	0.1209 (0.0000, 0.6245)	0.996	0.319	0.733
Duration with one robotic surgical instrument visible on the right side	3902.6350 (3041.1100, 5271.6125)	2434.1650 (1235.4575, 5244.2625)	1660.6750 (1170.2475, 3038.1175)	−2.330	0.020	0.655
Mean duration per minute with two robotic surgical instruments visible on the right side	11.1052 (8.4306, 20.5451)	16.7283 (8.1897, 24.2237)	17.4701 (6.3102, 32.1263)	1.246	0.213	0.710
Total clutch-use count	51.0000 (13.2500, 129.7500)	43.0000 (6.5000, 94.5000)	32.5000 (6.7500, 72.2500)	−1.166	0.244	0.710
Total camera-use duration	292.0900 (124.2375, 431.5400)	207.1050 (28.4775, 292.2600)	122.3050 (43.7675, 279.4225)	−1.592	0.111	0.655
Mean camera-use count per minute	0.6900 (0.5175, 1.2750)	0.6450 (0.3200, 1.0600)	0.6550 (0.4400, 0.8200)	−0.739	0.460	0.779
Total switching-use count	33.0000 (11.7500, 51.2500)	39.0000 (0.7500, 60.0000)	17.0000 (2.2500, 50.2500)	−0.463	0.644	0.910
Total switching-use duration	169.5200 (51.4550, 243.5525)	246.6900 (3.0225, 378.6150)	92.8050 (17.6025, 272.5975)	−0.266	0.790	0.942

Detailed summary table available in Supplementary Table S3. FDR: false discovery rate. All time-related metrics in the table are reported in seconds. The operative field was divided equally into left and right sides, and spatial distances were calculated with respect to the centers of the left and right halves.

## Data Availability

Data and code supporting the findings of this study may be obtained from the corresponding authors upon reasonable request.

## Supplementary Materials

The following supporting information can be downloaded at https://www.mdpi.com/article/10.3390/jcm15176786/s1: Supplementary Figure S1: Residual diagnostic plots for the final exploratory multivariable regression model in the full study sample (*n* = 42). (A) Normal P–P plot of standardized residuals. (B) Standardized residuals versus standardized predicted values; Supplementary Table S1: Baseline characteristics; Supplementary Table S2: Comparison of relevant metrics in a robotic training system across groups stratified by annual laparoscopic surgery volume; Supplementary Table S3: Comparison of operative performance metrics in a robotic training system between urology and general surgery groups stratified by annual laparoscopic surgery volume; Supplementary Table S4: Stepwise linear regression analysis of the association between GEARS score and operative metrics in robotic surgery; Supplementary Table S5: Inter-rater reliability of GEARS score within each rating batch; Supplementary Table S6: Sequential results of the stepwise multiple linear regression analysis; Supplementary Table S7: Specialty-exclusion sensitivity analysis of the exploratory multivariable model.

## Abbreviations

The following abbreviations are used in this manuscript:

GEARS	Global Evaluative Assessment of Robotic Skills
OSATS	Objective Structured Assessment of Technical Skills
FDR	False Discovery Rate
VIF	Variance Inflation Factor
