# Supplementary material for: Association Between Video-Derived Digital Operative Metrics and GEARS Scores in Robotic Surgery and Development of an Exploratory Multivariable Model
Source: J Clin Med. 2026 Sep 1;15(17):6786. doi: 10.3390/jcm15176786 (PMC13566548; doi:10.3390/jcm15176786)
Supplement: Supplementary file 1 [file jcm-15-06786-s001.zip › jcm-4431846-supplementary.pdf]

Article

Association Between Video-Derived Digital Operative Metrics and GEARS Scores in Robotic Surgery and Development of an Exploratory Multivariable Model

Xingyuan Xiao [1,2,3,†], Yuanzhuo Du [1,2,3,†], Zhenyu Liu [1,2,3,†], Xuewei Zhu [1], Kun Yang [1,2,3,*] and Bing Li [1,2,3,*]

[1] Department of Urology, Zhongnan Hospital of Wuhan University, Wuhan, 430071, China
[2] Hubei Key Laboratory of Urological Diseases, Wuhan, 430071, China
[3] Institute of Urology, Wuhan University, Wuhan, 430071, China
* Correspondence: jesuisking198411@hotmail.com (K.Y.); bingli2023@whu.edu.cn (B.L.)
† These authors contributed equally to this work.

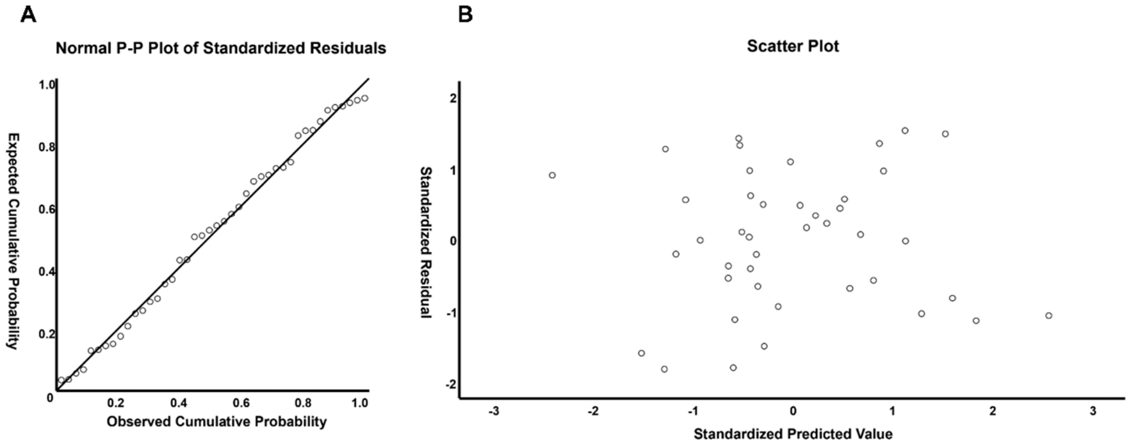

Supplementary Figure S1. Residual diagnostic plots for the final exploratory multivariable regression model in the full study sample (n = 42). (A) Normal P–P plot of standardized residuals. (B) Standardized residuals versus standardized predicted values.

Supplementary Table S1. Baseline Characteristics.

Robotic training system metrics	Annual laparoscopic surgery volume ≥200 (n=22)	Annual laparoscopic surgery volume ＜ 200 (n=20)	P-value	FDR

GEARS score based on surgical video recordings	24.4140±1.8576	24.6950±1.2292	0.563	0.808
Maximum distance from the left-side center point	0.5013(0.4853,0.5212)	0.5006(0.4846,0.5199)	0.821	0.935
Minimum distance from the left-side center point	0.0034(0.0019,0.0081)	0.0033(0.0018,0.0060)	0.696	0.881
Maximum leftward distance from the left-side center point	0.1833(0.1732,0.1935)	0.1910(0.1809,0.1958)	0.141	0.808
Maximum rightward distance from the left-side center point	0.2498(0.2497,0.2498)	0.2498(0.2496,0.2498)	0.966	0.988
Maximum upward distance from the left-side center point	0.4667(0.4592,0.4713)	0.4614(0.4348,0.4692)	0.314	0.808
Maximum downward distance from the left-side center point	0.4203±0.0189	0.4285±0.0208	0.187	0.808
Mean distance from the left-side center point	0.2064±0.0260	0.2072±0.0190	0.905	0.948
Maximum distance from the right-side center point	0.5050(0.4832,0.5189)	0.5086(0.4956,0.5198)	0.606	0.808
Minimum distance from the right-side center point	0.0041(0.0021,0.0090)	0.0033(0.0020,0.0057)	0.385	0.808
Maximum leftward distance from the right-side center point	0.2500(0.2498,0.2500)	0.2500(0.2496,0.2500)	0.483	0.808
Maximum rightward distance from the right-side center point	0.1863(0.1694,0.1907)	0.1832(0.1724,0.1897)	0.830	0.935
Maximum upward distance from the right-side center point	0.4648(0.4527,0.4695)	0.4662(0.4495,0.4742)	0.554	0.808
Maximum downward distance from the right-side center point	0.4227(0.4181,0.4339)	0.4255(0.4144,0.4389)	0.850	0.935
Mean distance from the right-side center point	0.2378±0.0283	0.2241±0.0335	0.158	0.808
Total number of instrument-out-of-view events	330.5900±224.7600	449.0500±319.3180	0.169	0.808
Mean number of instrument-out-of-view events per minute	3.7754±1.3585	4.1632±1.3056	0.352	0.808
Duration with one robotic surgical instrument visible on the left side	3849.9995±2139.2070	5101.8880±2880.8973	0.116	0.808

Mean duration per minute with one robotic surgical instrument visible on the left side	44.9435±7.0748	45.7092±5.5662	0.701	0.881
Duration with two robotic surgical instruments visible on the left side	551.4550(221.8700,1088.5125)	483.7200(188.2475,698.5625)	0.597	0.808
Mean duration per minute with two robotic surgical instruments visible on the left side	5.8899(4.0706,11.1738)	4.9167(2.5326,7.4275)	0.096	0.808
Duration with three robotic surgical instruments visible on the left side	16.9500(0.0000,56.4925)	5.8150(2.7800,11.2425)	0.464	0.808
Mean duration per minute with three robotic surgical instruments visible on the left side	0.1509(0.0000,0.5278)	0.0565(0.0276,0.1402)	0.479	0.808
Duration with one robotic surgical instrument visible on the right side	2324.6650(1303.3225,3038.1175)	3862.7850(1713.6625,5319.9775)	0.041	0.808
Mean duration per minute with one robotic surgical instrument visible on the right side	30.1847±10.2006	35.3605±7.0678	0.062	0.808
Duration with two robotic surgical instruments visible on the right side	1856.8200(373.4975,3372.7925)	1684.0550(322.7500,2619.1525)	0.420	0.808
Mean duration per minute with two robotic surgical instruments visible on the right side	19.6594±12.7436	12.7842±8.3470	0.044	0.808
Duration with three robotic surgical instruments visible on the right side	64.5100(4.4575,190.9375)	65.8350(4.6600,130.3550)	0.743	0.884
Mean duration per minute with three robotic surgical instruments visible on the right side	0.7644(0.1212,1.3462)	0.5192(0.0739,1.1076)	0.419	0.808
Total clutch-use count	39.5000(7.0000,72.2500)	51.0000(10.2500,109.0000)	0.392	0.808

Mean clutch-use count per minute	0.4050(0.2075,0.5965)	0.4250(0.2575,0.8100)	0.481	0.808
Total clutch-use duration	97.3950(15.3950,192.2275)	107.1450(24.7025,269.3900)	0.435	0.808
Mean clutch-use duration per minute	1.1150(0.4525,1.5875)	0.9000(0.5275,1.6950)	0.588	0.808
Mean duration per clutch use	2.1900(1.8875,2.4725)	2.1350(2.0125,2.3525)	0.880	0.944
Total camera-use count	55.5000(20.2500,88.5000)	82.0000(27.5000,153.2500)	0.231	0.808
Mean camera-use count per minute	0.6550(0.4025,0.8775)	0.6650(0.5125,1.2100)	0.554	0.808
Total camera-use duration	155.3850(43.7675,243.6300)	253.0.350(67.7200,436.2650)	0.190	0.808
Mean camera-use duration per minute	1.6100(1.0725,2.4000)	2.2150(1.3475,3.2475)	0.262	0.808
Mean duration per camera use	2.6600(2.2425,3.1950)	2.7050(2.3475,3.3850)	0.481	0.808
Total switching-use count	34.0000(3.7500,50.2500)	33.0000(2.7500,53.7500)	0.990	0.990
Mean switching-use count per minute	0.2786±0.2091	0.2420±0.1940	0.561	0.808
Total switching-use duration	167.5550(23.8375,275.1775)	169.5200(22.6100,259.4100)	0.743	0.884
Mean switching-use duration per minute	1.6986±1.3894	1.2540±0.9357	0.236	0.808
Mean duration per switch	5.5700(3.6475,7.2725)	4.3100(3.7900,6.6700)	0.419	0.808

FDR: False Discovery Rate. All time-related metrics in the table are reported in seconds. The operative field was divided equally into left and right sides, and spatial distances were calculated with respect to the centers of the left and right halves.

Supplementary Table S2. Comparison of Relevant Metrics in a Robotic Training System Across Groups Stratified by Annual Laparoscopic Surgery Volume.

Robotic training system metrics	Urology		P-value	FDR	General Surgery		P-value	FDR
	Annual laparoscopic surgery volume ≥200	Annual laparoscopic surgery volume ＜ 200			Annual laparoscopic surgery volume ＜ 200	Annual laparoscopic surgery volume ＜ 200		

GEARS score based on surgical video recordings	26.5250±1.0935	24.7600±1.1108	0.019	0.116	23.7670±1.9251	24.6300±1.3953	0.251	0.757
Maximum distance from the left-side center point	0.4721±0.0236	0.4972±0.0234	0.095	0.182	0.5081±0.0155	0.5011±0.0200	0.364	0.822
Minimum distance from the left-side center point	0.0098±0.0079	0.0048±0.0030	0.294	0.404	0.0029(0.0015,0.0048)	0.0027(0.0016,0.0051)	0.717	0.837
Maximum leftward distance from the left-side center point	0.1586±0.0376	0.1855±0.0158	0.074	0.182	0.1887±0.0095	0.1899±0.0088	0.758	0.855
Maximum rightward distance from the left-side center point	0.2493(0.2482,0.2497)	0.2498(0.2495,0.2498)	0.121	0.213	0.2498(0.2497,0.2498)	0.2498(0.2495,0.2498)	0.936	0.969
Maximum upward distance from the left-side center point	0.4178±0.0328	0.4501±0.0232	0.057	0.176	0.4690(0.4633,0.4713)	0.4669(0.4451,0.4711)	0.410	0.822
Maximum downward distance from the left-side center point	0.4151±0.0120	0.4281±0.0214	0.28	0.397	0.4186±0.0202	0.4289±0.0214	0.258	0.757
Mean distance from the left-side center point	0.1901±0.0355	0.1995±0.0089	0.636	0.718	0.2093±0.0239	0.2150±0.0234	0.584	0.837

Maximum distance from the right-side center point	0.4816(0.4711,0.4949)	0.5036(0.4910,0.5134)	0.090	0.1820	0.5141(0.5027,0.5215)	0.5126(0.4994,0.5211)	0.869	0.9400
Minimum distance from the right-side center point	0.0116(0.0073,0.0153)	0.0047(0.0026,0.0072)	0.048	0.1620	0.0025(0.0010,0.0042)	0.0020(0.0009,0.0041)	0.621	0.8370
Maximum leftward distance from the right-side center point	0.2498(0.2496,0.2500)	0.2500(0.2498,0.2500)	0.194	0.3050	0.2500(0.2500,0.2500)	0.2499(0.2492,0.2500)	0.079	0.7570
Maximum rightward distance from the right-side center point	0.1442±0.0723	0.1826±0.0087	0.367	0.4750	0.1842±0.0108	0.1802±0.0123	0.434	0.8300
Maximum upward distance from the right-side center point	0.3848(0.3300,0.4527)	0.4621(0.4176,0.4726)	0.090	0.1820	0.4665(0.4635,0.4719)	0.4662(0.4645,0.4759)	0.530	0.8370
Maximum downward distance from the right-side center point	0.4033±0.0217	0.4187±0.0274	0.335	0.4470	0.4258±0.0209	0.4244±0.0202	0.876	0.9400
Mean distance from the right-side center point	0.2138±0.0413	0.2078±0.0274	0.753	0.7890	0.2460±0.0236	0.2403±0.0321	0.640	0.8370
Total number of instrument-out-of-view events	110.7500±68.3930	335.4000±190.0900	0.043	0.158	436.3300±227.6780	562.7000±387.8400	0.353	0.822

Mean number of instrument-out-of-view events per minute	4.4000±1.1695	3.7848±1.5414	0.489	0.5980	3.9658±1.4010	4.5416±0.9508	0.283	0.7780
Duration with one robotic surgical instrument visible on the left side	1192.9275±501.8955	4740.7940±2437.4942	0.015	0.1100	4676.6617±1799.0072	5462.9820±3360.0867	0.518	0.8370
Mean duration per minute with one robotic surgical instrument visible on the left side	49.5185±2.5584	47.8104±4.1130	0.460	0.5780	42.4703±8.2104	43.6079±6.2193	0.723	0.8370
Duration with two robotic surgical instruments visible on the left side	107.2275±64.1984	438.5650±248.3653	0.002	0.0880	691.2050(399.9200,2061.1025)	463.8900(181.9675,1067.5825)	0.187	0.7570
Mean duration per minute with two robotic surgical instruments visible on the left side	3.4871(3.1420,6.6683)	5.3959(2.3989,6.7602)	0.777	0.7950	9.7592±6.9630	5.6646±3.8918	0.100	0.7570
Duration with three robotic surgical instruments visible on the left side	0.0000(0.0000,0.9975)	4.5100(2.6050,8.8875)	0.004	0.0880	22.5650(16.2400,182.6800)	6.6650(2.5275,73.5300)	0.209	0.7570
Mean duration per minute with three robotic surgical instruments visible on the left side	0.0000(0.0000,0.0278)	0.0617(0.0412,0.1291)	0.011	0.1060	0.2576(0.1507,1.3934)	0.0500(0.0200,0.8838)	0.234	0.7570
Duration with one robotic surgical instrument visible on the right side	939.9775±360.2780	3671.9500±1784.1713	0.012	0.1060	3242.2467±1550.0826	4034.7530±2802.0157	0.411	0.8220
Mean duration per minute with one robotic	39.5107±9.4592	38.0667±5.7402	0.7 7	0.	29.1547±8.5297	32.6543±7.4988	0. 3	0.

surgical instrument visible on the right side			28	781			24	822
Duration with two robotic surgical instruments visible on the right side	54.9600(47.4050,451.3225)	1053.6850(91.9200,1967.9350)	0.0090	0.1820	2549.0642±1702.8053	2206.2730±1622.3630	0.6336	0.8370
Mean duration per minute with two robotic surgical instruments visible on the right side	3.2206(2.0728,13.1370)	10.9626(2.4754,13.9908)	0.5772	0.6800	21.5060±11.8231	16.0553±8.4472	0.2337	0.7570
Duration with three robotic surgical instruments visible on the right side	0.0000(0.0000,3.9975)	43.3050(0.0000,102.4725)	0.0666	0.1820	79.9250(25.7650,168.2725)	99.0100(19.4625,217.7775)	0.9447	0.9690
Mean duration per minute with three robotic surgical instruments visible on the right side	0.0000(0.0000,0.1116)	0.3437(0.0000,0.7854)	0.0091	0.1820	0.7644(0.3949,1.2505)	0.5877(0.2302,1.2143)	0.6444	0.8370
Total clutch-use count	6.2500±0.9570	40.7000±38.9760	0.0211	0.1160	63.4200±49.2720	134.5000±138.1720	0.1550	0.7570
Mean clutch-use count per minute	0.3100±0.1257	0.3580±0.1974	0.6664	0.7300	0.5255±0.3861	0.9010±0.7000	0.1226	0.7570
Total clutch-use duration	15.0600(10.5200,18.7750)	60.1950(19.8200,153.8675)	0.0090	0.182	139.7967±101.6868	286.3150±297.7286	0.1166	0.757
Mean clutch-use duration per minute	0.7725±0.4472	0.7980±0.4989	0.9229	0.9	1.1492±0.7687	1.9750±1.4926	0.1110	0.7

				29				57
Mean duration per clutch use	2.3650±0.6444	2.1800±0.2170	0.611	0.708	2.1050(1.9150,2.4375)	2.1700(1.9850,2.4650)	0.717	0.837
Total camera-use count	13.0000(5.2500,17.7500)	78.0000(36.5000,142.0000)	0.024	0.117	68.5000(43.0000,89.5000)	89.5000(17.2500,167.7500)	0.717	0.837
Mean camera-use count per minute	0.5625±0.3531	1.076±0.8557	0.277	0.397	0.5500(0.3800,0.9575)	0.6200(0.4775,1.0675)	0.692	0.837
Total camera-use duration	24.5375±9.3376	257.9700±232.8841	0.011	0.106	217.6150(126.7350,259.0900)	306.0550(45.2950,586.1850)	0.598	0.837
Mean camera-use duration per minute	1.1400±0.4777	2.6120±1.8147	0.143	0.242	1.6700(1.0375,2.9675)	2.2150(1.3650,2.9825)	0.553	0.837
Mean duration per camera use	2.2850±0.5848	2.6390±0.4921	0.269	0.397	2.7000(2.3650,3.2700)	3.1650(2.5575,3.5925)	0.391	0.822
Total switching-use count	0.0000(0.0000,4.5000)	30.5000(6.0000,35.2500)	0.030	0.132	40.6700±20.7290	40.4000±36.4300	0.984	0.984
Mean switching-use count per minute	0.0000(0.0000,0.1275)	0.2050(0.0750,0.3375)	0.060	0.176	0.3850(0.2375,0.4825)	0.2700(0.0300,0.4575)	0.248	0.757

Total switching-use duration	0.0000(0.0000,32.3250)	135.1400(25.8675,210.6650)	0.043	0.158	240.2600±133.0020	193.3140±160.3762	0.461	0.837
Mean switching-use duration per minute	0.0000(0.0000,0.9375)	1.0250(0.3375,1.7600)	0.112	0.205	2.0625±1.0801	1.3860±1.0510	0.154	0.757
Mean duration per switch	0.0000(0.0000,5.3850)	4.5600(2.8200,6.0700)	0.192	0.305	5.7450(4.3850,7.4250)	4.1150(3.8025,7.0875)	0.222	0.757

FDR: False Discovery Rate. All time-related metrics in the table are reported in seconds. The operative field was divided equally into left and right sides, and spatial distances were calculated with respect to the centers of the left and right halves.

Supplementary Table S3. Comparison of Operative Performance Metrics in a Robotic Training System Between Urology and General Surgery Groups Stratified by Annual Laparoscopic Surgery Volume.

**Robotic training system metrics**	**Annual laparoscopic surgery volume <153**	**Annual laparoscopic surgery volume 154–246**	**Annual laparoscopic surgery volume≥247**	**Z score**	**P-value**	**FDR**
GEARS score based on surgical video recordings	24.8000(23.8750, 25.2000)	25.0000(22.3250, 25.6500)	25.0000(23.3000, 25.8000)	0.393	0.695	0.910
Maximum distance from the left-side center point	0.5006(0.4853,0.5149)	0.5068(0.4739,0.5208)	0.5007(0.4853,0.5231)	0.127	0.899	0.942
Minimum distance from the left-side center point	0.0040(0.0018,0.0053)	0.0027(0.0014,0.0055)	0.0048(0.0020,0.0116)	0.993	0.321	0.733
Maximum leftward distance from the left-side center point	0.1910(0.1854,0.1951)	0.1920(0.1757,0.1953)	0.1793(0.1710,0.1907)	-1.754	0.079	0.655

Maximum rightward distance from the left-side center point	0.2498(0.2497,0.2498)	0.2498(0.2490,0.2498)	0.2498(0.2497,0.2498)	-0.848	0.397	0.733
Maximum upward distance from the left-side center point	0.4618(0.4453,0.4698)	0.4646(0.4462,0.4693)	0.4688(0.4571,0.4714)	0.866	0.387	0.733
Maximum downward distance from the left-side center point	0.4306(0.4138,0.4368)	0.4276(0.4057,0.4500)	0.4241(0.4067,0.4291)	-1.235	0.217	0.710
Mean distance from the left-side center point	0.2007(0.1962,0.2178)	0.2001(0.1858,0.2310)	0.2076(0.1911,0.2282)	0.277	0.782	0.942
Maximum distance from the right-side center point	0.5086(0.4910,0.5221)	0.5113(0.4978,0.5199)	0.5045(0.4790,0.5153)	-0.842	0.400	0.733
Minimum distance from the right-side center point	0.0026(0.0018,0.0052)	0.0040(0.0019,0.0078)	0.0046(0.0023,0.0090)	1.224	0.221	0.710
Maximum leftward distance from the right-side center point	0.2500(0.2498,0.2500)	0.2500(0.2497,0.2500)	0.2499(0.2498,0.2500)	-0.219	0.827	0.942
Maximum rightward distance from the right-side center point	0.1830(0.1700,0.1861)	0.1900(0.1817,0.1963)	0.1726(0.1613,0.1888)	-0.381	0.703	0.910
Maximum upward distance from the right-side center point	0.4655(0.4523,0.4752)	0.4672(0.4186,0.4713)	0.4641(0.4527,0.4679)	-0.969	0.332	0.733
Maximum downward distance from the right-side center point	0.4292(0.4182,0.4403)	0.4243(0.4148,0.4437)	0.4211(0.4116,0.4317)	-1.328	0.184	0.710
Mean distance from the right-side center point	0.2211(0.1848,0.2463)	0.2377(0.2157,0.2590)	0.2449(0.2208,0.2583)	1.523	0.128	0.655
Total number of instrument-out-of-view events	408.0000(236.7500,691.0000)	320.5000(180.5000,575.2500)	296.5000(104.5000,417.7500)	-1.523	0.128	0.655
Mean number of instrument-out-of-view events per minute	4.1106(2.9920,5.007)	3.6068(3.0179,4.9594)	3.3584(2.8472,5.0229)	-0.392	0.695	0.910
Duration with one robotic surgical instrument visible on the left side	5720.7500(4006.8450,7392.3175)	4582.4650(1365.4950,7112.0825)	3656.3200(1455.3200,5147.5475)	-1.915	0.055	0.655

Mean duration per minute with one robotic surgical instrument visible on the left side	46.7391(41.9406, 49.8189)	46.1104(39.5074, 53.1189)	46.6157(41.2434, 49.6859)	-0.138	0.890	0.942
Duration with two robotic surgical instruments visible on the left side	579.0500(201.4175,715.5550)	422.5850(146.7225,1438.3400)	428.6000(221.8700,775.9425)	-0.046	0.963	0.963
Mean duration per minute with two robotic surgical instruments visible on the left side	4.9167(2.3684,7.2721)	5.4866(3.3492,11.1738)	5.8899(4.4559,9.9936)	1.707	0.088	0.655
Duration with three robotic surgical instruments visible on the left side	6.6650(2.8650,10.3675)	11.6850(2.2300,78.4450)	13.7300(0.0000,44.5675)	0.602	0.547	0.860
Mean duration per minute with three robotic surgical instruments visible on the left side	0.0503(0.0222,0.0741)	0.1482(0.0294,0.7671)	0.1209(0.0000,0.6245)	0.996	0.319	0.733
Duration with one robotic surgical instrument visible on the right side	3902.6350(3041.1100,5271.6125)	2434.1650(1235.4575,5244.2625)	1660.6750(1170.2475,3038.1175)	-2.330	0.020	0.655
Mean duration per minute with one robotic surgical instrument visible on the right side	36.7311(29.3909, 42.1294)	33.8905(26.7548, 38.5052)	30.8427(19.8370, 39.3391)	-1.500	0.134	0.655
Duration with two robotic surgical instruments visible on the right side	1838.2000(371.8100,2574.3375)	1899.6850(361.0875,3326.0375)	1577.2950(269.6225,3590.8200)	0.254	0.800	0.942
Mean duration per minute with two robotic surgical instruments visible on the right side	11.1052(8.4306,20.5451)	16.7283(8.1897,24.2237)	17.4701(6.3102,32.1263)	1.246	0.213	0.710
Duration with three robotic surgical instruments visible on the right side	65.8350(6.0600,121.1600)	96.5200(3.9975,223.9675)	45.2600(1.7125,195.1400)	0.208	0.835	0.942

Mean duration per minute with three robotic surgical instruments visible on the right side	0.4848(0.0981,0.7854)	0.7350(0.1116,1.3462)	0.7928(0.0329,1.3877)	0.902	0.367	0.733
Total clutch-use count	51.0000(13.2500,129.7500)	43.0000(6.5000,94.5000)	32.5000(6.7500,72.2500)	-1.166	0.244	0.710
Mean clutch-use count per minute	0.4250(0.2550,0.8400)	0.4000(0.2075,0.6875)	0.4400(0.2000,0.5965)	-0.646	0.518	0.844
Total clutch-use duration	107.1450(32.0450,305.8900)	99.0950(13.1000,185.5150)	76.7900(15.0925,193.3375)	-1.131	0.258	0.710
Mean clutch-use duration per minute	0.9000(0.6775,1.7400)	0.9800(0.4850,1.4250)	1.1150(0.3875,1.5875)	-0.773	0.440	0.774
Mean duration per clutch use	2.1600(2.0775,2.4650)	2.1000(1.9200,2.3700)	2.1600(1.8750,2.5250)	-0.900	0.368	0.733
Total camera-use count	111.0000(45.500,156.0000)	66.5000(15.0000,95.5000)	45.0000(20.2500,91.2500)	-1.650	0.099	0.655
Mean camera-use count per minute	0.6900(0.5175,1.2750)	0.6450(0.3200,1.0600)	0.6550(0.4400,0.8200)	-0.739	0.460	0.779
Total camera-use duration	292.0900(124.2375,431.5400)	207.1050(28.4775,292.2600)	122.3050(43.7675,279.4225)	-1.592	0.111	0.655
Mean camera-use duration per minute	2.1500(1.3625,3.1375)	1.9300(0.9975,2.9825)	1.5100(1.0025,2.6125)	-1.165	0.244	0.710
Mean duration per camera use	2.6300(2.3600,3.1225)	3.1200(2.3000,3.5675)	2.6600(2.1875,2.8550)	-0.462	0.644	0.910
Total switching-use count	33.0000(11.7500,51.2500)	39.0000(0.7500,60.0000)	17.0000(2.2500,50.2500)	-0.463	0.644	0.910
Mean switching-use count per minute	0.2150(0.1150,0.3600)	0.3450(0.0225,0.4900)	0.2100(0.0675,0.4375)	-0.093	0.926	0.948

Total switching-use duration	169.5200(51.4550,243.5525)	246.6900(3.0225, 378.6150)	92.8050(17.6025, 272.5975)	-0.266	0.790	0.942
Mean switching-use duration per minute	1.0250(0.6075,2.2350)	1.9150(0.0900,2.8025)	0.9900(0.2175,2.7300)	0.173	0.862	0.942
Mean duration per switch	4.7500(3.8500,7.2700)	5.2450(2.9625,7.1500)	4.8100(2.1450,7.2425)	-0.405	0.686	0.910

FDR: False Discovery Rate. All time-related metrics in the table are reported in seconds. The operative field was divided equally into left and right sides, and spatial distances were calculated with respect to the centers of the left and right halves.

Supplementary Table S4. Stepwise Linear Regression Analysis of the Association Between GEARS Score and Operative Metrics in Robotic Surgery.

Model	R^2	F	P
GEARS Score=25.242 [95%CI:24.658~25.826]-0.001 [95%CI:-0.002~0.000]*Duration with two robotic surgical instruments visible on the left side	0.243	12.839	＜0.001
GEARS Score=24.659 [95%CI:23.915~25.403]-0.001 [95%CI:-0.001~0.000]*Duration with two robotic surgical instruments visible on the left side+0.622 [95%CI:0.092~1.152]*Mean camera-use count per minute	0.338	9.975	＜0.001
GEARS Score=23.595 [95%CI:22.567~24.622]-0.001 [95%CI:-0.001~0.000]*Duration with two robotic surgical instruments visible on the left side+0.824 [95%CI:0.314~1.334]*Mean camera-use count per minute+140.003 [95%CI:39.435~240.572]*Minimum distance from the left-side center point	0.453	10.481	＜0.001
GEARS Score=32.141 [95%CI:23.667~40.616]+0.863 [95%CI:0.371~1.354]*Mean camera-use count per minute+104.389 [95%CI:1.589~207.189]*Minimum distance from the left-side center point-17.074 [95%CI:-33.890~-0.259]*Maximum distance from the right-side center point	0.509	9.588	＜0.001

All time-related metrics in the table are reported in seconds. The operative field was divided equally into left and right sides, and spatial distances were calculated with respect to the centers of the left and right halves. The Durbin–Watson statistic for the final model was 2.183. For transparent reporting of the fitted regression coefficients, the sample-specific fitted regression equation was:

GEARS Score=32.141+0.863*Mean camera-use count per mi-nute+104.389*Minimum distance from the left-side center point-17.074*Maximum distance from the right-side center point.

Supplementary Table S5. Inter-rater reliability of GEARS score within each rating batch.

Rating batch	Number of participants	Number of raters	ICC	95% Confidence interval
Batch 1	14	7	0.601	0.250-0.841
Batch 2	14	7	0.709	0.421-0.887
Batch 3	14	7	0.638	0.251-0.865

ICC: Average-measure intra-class correlation coefficient.

Supplementary Table S6. Sequential Results of the Stepwise Multiple Linear Regression Analysis.

Step	Variable	Unstandardized coefficients		Standardized coefficient	t	P value	95% CI for B		Collinearity statistics	
		B	SE				Lower	Upper	Tolerance	VIF
1		25.242	0.289		87.333	0	24.658	25.826		
	Duration with two robotic surgical instruments visible on the left side	-0.001	0	-0.493	-3.583	0.001	-0.002	0	1	1
2		24.659	0.368		67.047	0	23.915	25.403		
	Duration with two robotic surgical instruments visible on the left side	-0.001	0	-0.462	-3.531	0.001	-0.001	0	0.99	1.01
	Mean camera-use count	0.622	0.262	0.31	2.372	0.023	0.092	1.152	0.99	1.01

	per minute									
3		23.595	0.508		46.491	0	22.567	24.622		
	Duration with two robotic surgical instruments visible on the left side	-0.001	0	-0.318	-2.426	0.02	-0.001	0	0.839	1.192
	Mean camera-use count per minute	0.824	0.252	0.411	3.269	0.002	0.314	1.334	0.91	1.099
	Minimum distance from the left-side center point	140.003	49.678	0.377	2.818	0.008	39.435	240.572	0.803	1.246
4		32.141	4.182		7.685	0	23.667	40.616		
	Duration with two robotic surgical instruments visible on the left side	0	0	-0.227	-1.706	0.096	-0.001	0	0.747	1.338
	Mean camera-use count per minute	0.863	0.243	0.431	3.556	0.001	0.371	1.354	0.905	1.106
	Minimum distance from the left-side center point	104.389	50.736	0.281	2.058	0.047	1.589	207.189	0.709	1.41

	Maximum distance from the right-side center point	-17.074	8.299	-0.284	-2.057	0.047	-33.89	-0.259	0.696	1.436

VIF: variance inflation factor.

Supplementary Table S7. Specialty-Exclusion Sensitivity Analysis of the Exploratory Multivariable Model.

Step	Variable	Unstandardized coefficients		Standardized coefficient	t	P value	95% CI for B		Collinearity statistics	
		B	SE				Lower	Upper	Tolerance	VIF
1		25.297	0.325		77.864	0	24.636	25.957		
	Duration with two robotic surgical instruments visible on the left side	-0.001	0	-0.493	-3.302	0.002	-0.002	0	1	1
2		24.71	0.404		61.11	0	23.887	25.533		
	Duration with two robotic surgical instruments visible on the left side	-0.001	0	-0.465	-3.278	0.002	-0.001	0	0.992	1.008
	Mean camera-use count per minute	0.624	0.279	0.317	2.233	0.032	0.055	1.192	0.992	1.008
3		23.68	0.559		42.33	0	22.54	24.819		
	Duration with two	-0.001	0	-0.319	-2.212	0.034	-0.001	0	0.828	1.207

	robotic surgical instruments visible on the left side									
	Mean camera-use count per minute	0.799	0.269	0.405	2.969	0.006	0.251	1.347	0.924	1.082
	Minimum distance from the left-side center point	139.153	55.951	0.365	2.487	0.018	25.185	253.121	0.799	1.252
4		31.843	4.594		6.931	0	22.474	41.213		
	Duration with two robotic surgical instruments visible on the left side	0	0	-0.232	-1.572	0.126	-0.001	0	0.739	1.353
	Mean camera-use count per minute	0.84	0.261	0.426	3.215	0.003	0.307	1.373	0.917	1.09
	Minimum distance from the left-side center point	108.977	56.686	0.286	1.922	0.064	-6.635	224.589	0.728	1.373
	Maximum distance from the right-side center	-16.36	9.142	-0.267	-1.789	0.083	-35.006	2.286	0.723	1.384

point	

VIF: variance inflation factor.
